# Telocytes and inflammation: A review

**DOI:** 10.1097/MD.0000000000035983

**Published:** 2023-11-17

**Authors:** Yuhua Zhang, Hu Tian

**Affiliations:** a The First Clinical Medical College, Shandong University of Traditional Chinese Medicine, Department of General Surgery, Shandong Provincial Qianfoshan Hospital, Jinan, Shandong, China; b Department of General Surgery, The First Affiliated Hospital of Shandong First Medical University & Shandong Provincial Qianfoshan Hospital, Key Laboratory of Metabolism and Gastrointestinal Tumor, Jinan, Shandong, China.

**Keywords:** inflammation, interstitial cells of Cajal, telocytes, telopodes

## Abstract

Telocytes are a new type of interstitial cell with a diverse morphology and important functions, such as mechanical support, signal transduction, immune regulation, and tissue repair. In this paper, the origin and physiological and pathological functions of telocytes as well as their role in inflammation will be discussed, and the functions and targets of telocytes in inflammation will be fully reviewed, which may contribute to a new therapeutic strategy for inflammatory diseases in the future.

## 1. Introduction

**Figure 1.**
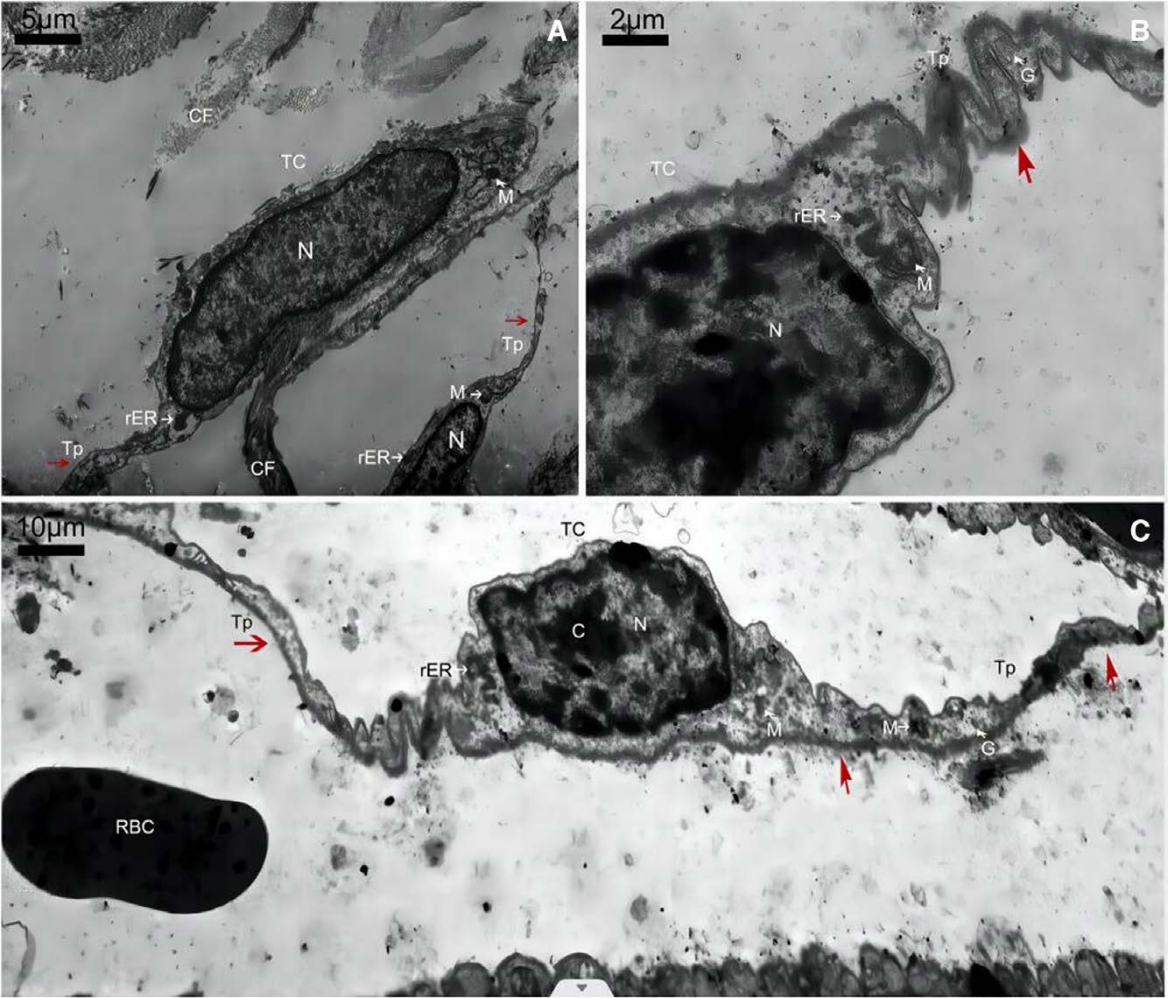
Ultrastructure of telocytes in the atherosclerotic carotid artery. The scale bars are 5 μm (A) and 2 μm (B) and 10 μm (C). CF = collagenous fiber; L = liposome; M = mitochondria; N = nucleus; rER = rough endoplasmic reticulum; TC = telocyte; Tp = telopode.

Telocytes (TCs), which are of mesenchymal origin and are resident connective tissue (stromal) cells, were first discovered by Prof L.M. Pescu in 2005 under transmission electron microscopy (TEM). Their structures are similar to those of interstitial cells of Cajal (ICCs), and they were called interstitial Cajal-like cells. After further study, researchers found that TC markers were obviously different from ICC molecular markers, so they were renamed telocytes. TCs are widely found in several mammalian tissues and organs, and their cytosomes are diverse, with dendritic, ellipsoidal, pear-shaped and polygonal forms predominating. TEM has revealed TCs with elongated cytoplasmic protrusions called telopods (TPs), and each TC has one or several TPs (usually 2 or 3). The TPs are not uniform in their thickness all along and show narrow zones (podomers) and thickenings (podomes)^[1]^ (Fig. 1^[2]^). TCs form homocytic or heterocytic contacts through TPs, thereby forming three-dimensional network structures and transmitting signaling molecules through prosecretory and/or paracrine binding to enter the vascular, neural and endocrine systems by the outgrowth and shedding of vesicles. In this interaction, organelles, mRNA, microRNA, long-stranded noncoding RNA and genomic DNA are subsequently transferred to play a role in signal transmission.^[3]^ In addition to signaling, TCs also participate in various physiological and pathological processes, such as the immune response, inflammatory response, angiogenesis and invasion and metastasis of cancer cells. Besides, TCs were shown to function as crucial components of the stem cell niche.^[4]^ Telocytes may become an effective means of clinical treatment of inflammatory diseases, malignant tumors and other diseases.

## 2. Origin and physiological role of telocytes

TCs are widespread in a variety of tissues and organs, such as cardiac tissues,^[5,6]^ vessels,^[7]^ the trachea and lungs,^[8,9]^ gallbladder,^[10]^ liver,^[11]^ Intestinal tissues,^[12]^ duodenum,^[13]^ colon,^[14]^ and spleen,^[15]^ and they are also found in the male genitals including the testis,^[16]^ and epididymis,^[17]^as well as the female genital organs and the uterus,^[18,19]^ oviduct,^[20]^ and placenta.^[21]^ In addition, TCs are also found in the pleura,^[22]^ skin,^[1]^ parotid glands,^[23]^ skeletal muscle,^[24,25]^ and epithelium.^[26]^ After more than 20 years of exploration, TCs have been shown to exist in almost all tissues and organs.^[27]^

Although TCs are distributed in the digestive, cardiovascular, respiratory, nervous, reproductive and urinary systems, as well as in the skin and skeletal muscle, there are tissue differences in the molecular markers they express, even within the same tissue^[4]^ For example, in human myocardial tissue, TCs are negative for nestin, CD13 and S-100 immunohistochemical indicators, but are positive for CD34, CD117, vimentin, and epithelial cell growth factor immunohistochemical indicators.^[28]^ In addition, TCs positive for CD34, C-Kit, and vimentin expression were found in human aortic, mitral, and tricuspid valves, which can form a 3D scaffold network within the heart valves and provide mechanical support. Moreover, as a major component of the endocardial “blood-heart barrier,” TCs participate in the cellular network and play a role in signaling, promoting myocardial repair and regeneration and immune surveillance, in addition to mechanical support.^[29,30]^ In human bladder tissue, 3 subtypes of TCs are distinguished by immunohistochemistry, and the subtypes differ in their expression of the same immunohistochemical markers in the same tissue. The subtypes of TCs in the submucosa and detrusor of the bladder are positive for CD34 and negative for α-smooth muscle actin, platelet-derived growth factor receptor alpha (PDGFR-α) and CD117.^[31]^ In the respiratory system, TCs are mainly distributed among the basement membrane of the fine bronchial epithelium, clusters of airway smooth muscle cells in the muscular layer, respiratory epithelium at the bronchoalveolar junction, and clusters of peripheral vessels, nerves, and stem cells, and in the above tissues, TCs show positive expression of CD34 and C-Kit and play a role in cellular communication and structural support.^[32]^ In addition, TCs express different positive biomarkers in the reproductive and digestive systems as well as in the skeleton and skin. A study found that miRNA may be a means to differentially discriminate TCs from other cells, and miR-193 was shown to be differentially expressed between TCs and other interstitial cells. In addition, reseachers have found that cardiomyocytes and other muscle cell-specific miRs (miR-133a, and miR-208a) are absent in TCs, which confirms the specificity of TCs and may be an important method to differentiate them from other interstitial cells.^[33]^ Although no specific molecular markers for TCs have been identified, the commonly used molecular markers are summarized as follows: C-Kit, CD34, CD117, vimentin, PDGFR-α, caveolin-1, vascular endothelial growth factor, inducible nitric oxide synthase, and so on.^[34–37]^

TCs are involved in tissue renewal, mechanical support, and immune modulation,^[38]^ and their specific role depends on different tissue distributions. In the cardiovascular system: because of the presence of a specific electrochemical gradient barrier exists in the endocardium, that is, the blood–brain barrier, and TCs are an important component of this barrier and are involved in the formation of a three-dimensional myocardial network and in intercardiac signaling and myocardial repair.^[39]^ TCs are able to support myocardial tissue and adult heart redevelopment as support cells^[40]^ and are the connecting and communicating cells of cardiac myocytes.^[41]^ In the respiratory system, TPs establish interstitial synapses with mast cells and participate in the regulation of tracheal secretion and contraction together with mast cells. In addition, ectopic pacing of the heart mainly originates from the myocardial sleeve of the pulmonary vein (PV), and TCs, as potential mechanoreceptors in the PV, can affect the pacing of the heart by sensing changes in PV pressure that trigger changes in nonselective cation currents and Cl^-^ currents in cardiomyocytes.^[42]^ The number of TCs in the lung is constantly changing during development from the fetal stage to adulthood, during which TCs are involved in lung angiogenesis, the formation of the air-blood barrier and lung tissue growth and development.^[43]^ In the digestive system, TCs not only participate in the construction of the three-dimensional network and the formation of the microenvironment, but also influence the contraction of smooth muscle and the movement of the gallbladder.^[44,45]^ In the reproductive system, TCs are present in the middle layer of the myometrium, which is considered the regulatory center of the myometrium and is responsible for receiving signals from the autonomic nerves and transmitting them to the cells of the myometrium through TCs; and studies have been performed on the release of certain stimulating factors by TCs that act on the myofibroblasts during the contraction of the uterus, thus causing the ion-activated channels in the myofibroblasts to function and causing muscle contraction, thereby participating in the regulation of blood flow and muscle contraction in the myometrium.^[46]^ In the urinary system, TCs are not only involved in the urinary reflex of the bladder and the dilatory movement of the bladder wall, thereby maintaining the normal three-dimensional structure of the testes, but they are also an important component of the blood-testis barrier and are involved in the regulation of spermatogenesis.^[47]^ In skin and skeletal muscle, TCs can integrate skeletal muscle fiber regulation and affect the regeneration of skin and skeletal muscle.^[25,48,49]^

## 3. Pathological role of telocytes

TCs act as a network organization for building the three-dimensional structure of tissues, providing a “scaffold-like” structure for tissue remodeling, providing mechanical support, mediating cell signaling, directing and caring for immature cells during organogenesis, and serving as a precursor for many mesenchymal-derived cells during adulthood. In addition, TCs are involved in regulating the progression of many diseases and are key players in the regeneration and repair of many organs.^[50]^ In the field of oncology research, TCs are involved in the formation of PDGFR-α mutation-associated gastrointestinal tumors. During the formation of these tumors, proteomic methods and gene sequence mutation detection have confirmed that TCs show reduced numbers, morphological changes and even nuclear sequence destruction during the formation of gastrointestinal mesenchymal tumors.^[51]^ Although this study did not find a clear pathogenesis of TCs in the process of tumor formation, it gives us important hints for the next steps in studying tumor formation. In the interstitial matrix between liver parenchymal cells of human liver tissues, TCs are heterogeneous, and alterations in their function are obvious, especially when liver fibrosis, liver tumors and inflammation occur, suggesting that TCs play an important role in the development of liver diseases.^[52]^ In a study on hepatocellular carcinoma (HCC), the expression of matrix metalloproteinase-9 in TCs was found to promote the metastasis of HCC. It was found that PDGF-α secreted by HCC activated the Ras/ERK signaling pathway in TCs and increased the expression of matrix metalloproteinase-9, which in turn accelerated the invasive metastasis of HCC.^[53,54]^ There is a large distribution of TCs and TPs between the submucosa and smooth muscle cells in bladder cancer. However, TCs and TPs are scarce in the neurogenic bladder. In addition, it was found that TCs in normal tissues with ezrin expression may compete for invasiveness and may be an indicator that can be used to reduce the recurrence rate of bladder cancer. Thus, TCs and ezrin may serve as predictors of bladder cancer invasiveness and recurrence.^[31]^ In fibrosis, when pathological loss occurs, the number of fibroblasts increases, producing large amounts of extracellular matrix (ECM) that disrupts tissue integrity. A study showed that TCs can synthesize ECM in response to hormonal stimulation and can interact with neighboring cells to produce exosomes and transmit intercellular biological signals.^[55,56]^ TCs have a potential role in the development of systemic fibrotic diseases and are important for improving the therapeutic outcome of related diseases. In a study of lymphedema, transplantation of TCs into lymphedema-damaged tissues promoted regeneration of the damaged tissues.^[38]^ It has been shown that TCs not only promote fibrosis reversal but also increase the number of TCs expressing the immune marker CD34+. Promoting the survival and growth of TCs by stimulating them in new ways or using new drugs may be a therapeutic strategy for the treatment of lymphedema fibrosis. In the field of dermatology, TCs are closely associated with or in contact with fibroblasts, papillary cells, adipocytes, collagen bands and elastic fibers, and their density and distribution are influenced by pathological conditions of the skin, such as psoriasis and systemic sclerosis.^[27,57]^ In addition, normalization of TC numbers is associated with disease remission and is involved in the process of skin tissue repair and revascularization.^[58,59]^ In a rat experiment on myocardial infarction, TCs were found to inhibit myocardial microvascular endothelial cell apoptosis by targeting Cdip1 silencing through exosomal miR-21-5p to inhibit myocardial infarction angiogenesis.^[60,61]^ In addition to affecting angiogenesis, TCs show strong immunoreactivity toward APG5, transforming growth factor beta and nuclear factor erythroid-2-related factor 2; affect phagocytosis and autophagy; and may also be involved in cell differentiation and regeneration.^[62]^ Endometriosis involves the survival and growth of glands and stroma outside the uterine cavity and is a common benign inflammatory gynecological disease. TCs play a potential role in endometriosis by affecting the inflammatory response, invasion and angiogenesis and may be a potential therapeutic option for endometriosis.^[63]^

TCs are involved in a range of inflammatory responses in addition to a variety of pathological processes, such as cancer cell invasion and metastasis, vascular regeneration, and fibrosis reversal.^[64]^ A report has found a significant increase in the TC concentration in tissues of chronically injured patients compared to healthy controls, which reduced the delay in wound healing, and the inflammatory response induced by LPS can be mediated by inflammatory inhibition, thereby limiting apoptosis, promoting skin cell proliferation and migration, and accelerating inflammatory repair. The following is a comprehensive description of the inflammatory response involving TCs.^[65]^

## 4. Inflammation and telocytes

The development and activity of the immune system is coordinated by cytokines, which are small, short-lived proteins necessary for paracrine, autocrine and endocrine signaling. TCs have their own cytokine profile and may influence the secretion of macrophages and B cells. The development of inflammation is the result of complex interactions between immune and nonimmune cells, tissue resident cells, and mesenchymal cells. TCs, as mesenchymal cells newly discovered in recent years, can prevent the abnormal activation of immune cells to control chronic inflammation and are active players in immune regulation and immune surveillance.^[66]^ Evidence has indicated that TCs may help prevent abnormal activation of immune cells and fibroblasts, attenuate changes in stromal tissue during fibrosis, promote regeneration and prevent the evolution of irreversible tissue damage. In the near future, TCs may offer new therapeutic opportunities to control the development of chronic inflammatory and fibrotic diseases.

### 4.1. Cardiac and cerebrovascular inflammation

TCs can affect the activity of immune cells through direct heterocellular connections or indirect paracrine effects and thus participate in local inflammatory processes or immune responses. A study on acute myocardial infarction in rats found severe TC death in the infarcted area of the heart, with a significant reduction in TC numbers.^[67,68]^ Moderate exercise in early myocardial infarction increases the number of myocardial TCs in the marginal zone but not in the infarct zone and improves postinfarction angiogenesis, fibrosis, and ventricular remodeling.^[69]^ Therefore, new strategies to effectively limit myocardial cell and TC death in the infarct zone are essential for myocardial infarct regeneration.

Experimental autoimmune encephalomyelitis (EAE) is a common inflammatory disease of the brain, and the expression of CD34+ in TCs was found to be significantly upregulated in the spleen of mice with EAE, and CD34+ TCs were shown to be involved in the regulation of immune response after spleen injury, specifically by recruiting of macrophages and proliferating stem cells and promotings tissue repair and regeneration in mice with EAE. Therefore, combined transplantation of TCs and stem cells may be a promising therapeutic strategy for the treatment and prevention of multiple autoimmune and chronic inflammatory diseases.^[15]^

### 4.2. Pulmonary inflammation

In the lungs, TCs are mainly distributed in the alveolar wall within the capillaries and are partially located in the interstitial space.Th1/Th2 imbalance is thought to be the pathogenesis of asthma. It has been found that TCs can affect the Th1/Th2 balance by downregulating the expressing of the Th2-related cytokines interleukin-4 and GATA binding protein 3 and Th2 cell differentiation and upregulating the expressing of the Th1-related cytokines IFN-γ and T-bet.^[70]^ TCs may promote the migration of mesenchymal Schwann cells (MSCs) to lung tissue, the survival of MSCs in lung tissue, and the therapeutic effects of MSCs on asthma by altering inflammatory cells and mediators.^[71]^ The treatment of experimental asthma by TCs was found to increase systemic and local transforming growth factor beta production and to alter the ratio of Treg cells.^[9]^ The interaction between TCs and Treg cells may be a key part of the cellular mechanism by which TCs ameliorate allergen-induced airway inflammation and hyperresponsiveness. Intravenously transplanted TCs significantly inhibit airway inflammation and airway hyperresponsiveness.

TCs in the lung have a specific connection with air–liquid epithelial cells, tolerance to movement and pressure, and flexibility among barriers. Dongli Song successfully constructed a mouse lung TC cell line that was clearly able to maintain the biological properties and behavior of primary TCs and to respond to lipopolysaccharide (LPS)-induced inflammatory responses. With LPS as a stimulus for infection and TNFα as an inflammatory mediator, the proliferative capacity of TCs was found to decrease with increasing LPS/TNFα concentration.^[72]^ Experiments have shown that TCs can maintain lung anatomical structure and function and provide a new alternative for the treatment of lung diseases.

### 4.3. Gastrointestinal inflammation

Inflammatory bowel disease, including Crohn disease and ulcerative colitis (UC), is a widespread autoimmune disease. In inflammatory bowel disease, inflammation tends to evolve into tissue fibrosis, which is closely related to the distribution and site of inflammation.^[73]^ There is evidence that prolonged exposure of intestinal fibroblasts to inflammatory mediators may contribute to their conversion to activated myogenic cells expressing α-smooth muscle actin, leading to abnormal collagen production and tissue remodeling. However, inflammation also appears to play a secondary role in fibrotic progression, so anti-inflammatory therapy may not limit intestinal fibrosis once the ECM is overdeposited.^[74]^ Notably, the number of TCs and TPs is negatively correlated with the amount of mature fibrillar collagen and is positively correlated with degraded collagen.^[56]^ A significantly reduced TC abundance was found in the mucosal myenteric and submucosal layers of the colonic wall in early and advanced UC. A normal distribution of TCs was observed in the intrinsic muscular layer in early UC, but TCs were reduced or even completely absent in the fibrotic area of the muscular layer and around the myenteric ganglion in advanced UC.^[75]^ Excessive deposition of ECM and the progressive reduction of TCs may alter the spatial relationships of TPs with neighboring immune cells, fibroblasts, smooth muscle cells, and neural structures, impairing intercellular signaling and function. Progressive loss of TCs in the intestinal wall leads to alterations in the three-dimensional organization of the ECM, which may accelerate the fibrotic process. In the intestine, the three-dimensional network of TCs is also thought to play specific mechanical and supportive roles in different layers of the intestinal wall, with resistance and deformability of the network affecting intestinal motility.^[50,76,77]^ More importantly, in the lamina propria, TCs and ICCs may form interconnected networks around smooth muscle bundles and myenteric plexus ganglia, and the reduction in TC and ICC networks within the neuromuscular compartment of the intestinal wall may largely contribute to the gastrointestinal motility disorders in patients with Crohn disease and UC.^[78]^

### 4.4. Skin inflammation

Systemic sclerosis (SSc) is a chronic connective tissue disease. In the early stages of SSc, the main histopathological features of the skin are perivascular inflammatory infiltration, dermal edema, and varying degrees of ECM accumulation in the papillary and reticular dermis.^[79,80]^ The late stage of SSc mostly shows severe fibrotic skin changes, irregular tight accumulation of collagen bundles, loss of capillaries, occlusion of small arteries and nerve fiber damage.^[80]^ In a study on the involvement of TCs in skin injury diseases, researchers found that TCs inhibit the release of inflammatory factors and promote the migration of epithelial cells in injured skin tissues, and this effect is achieved by influencing TC tropism through the LPS-related protein signaling pathway.^[34,65]^ TCs are normally present around perivascular inflammatory infiltrates in the reticular dermis but are absent when these infiltrates are located in the papillary dermis. It is also hypothesized that TCs may be involved in maintaining local tissue homeostasis by controlling fibroblast/myocyte activity. Future studies could focus on typing perivascular and perivascular TCs distributed in the reticular dermis in the disease state and assessing the possible role of TCs in preserving and regulating inflammatory cells in the perivascular space.^[81–83]^

Psoriasis is also a common inflammatory skin disease that is mainly considered a keratosis with a genetic predisposition. In psoriasis, TEM shows the presence of apoptotic distal cells with nuclei, dystrophic distal cells with broken TPs, and even TCs with nuclear extrusion, membrane disintegration, and cytoplasmic breakage. It was found that in small vessels that have lost the protective membrane formed by TCs, the phenotype of the vascular smooth muscle cells undergoes profound changes.^[84]^ Therefore, it has been suggested that loss of perivascular TCs may be important in the characteristic vascular pathology of psoriasis.

### 4.5. Gynecological inflammation

Endometriosis, pelvic inflammatory disease and tubal inflammatory disease are the most common gynecological inflammatory diseases and are important causes of female infertility. Studies have shown that immune cells such as monocytes and macrophages play an important role in the local peritoneal immune response in endometriosis or pelvic inflammatory disease, and their dysfunction or abnormal changes in their numbers may alter smooth muscle movement and the microcirculation in endometriosis, leading to pelvic pain and even infertility.^[85,86]^ As novel mesenchymal cells, TCs play a crucial role in promoting angiogenesis and increasing endometrial mesenchymal and inflammatory cell activity. Moreover, TCs can be involved in the structural and functional abnormalities of the fallopian tubes, specifically the changes in diseased tubal tissues, such as those of the aseptic inflammatory disease endometriosis and infectious acute salpingitis. In the tubal tissues of acute salpingitis, multiple ultrastructural impairments in TC bodies and TPs are seen, accompanied by significant TC loss and elevated collagen content. Similarly, TC damage was also found in the tubal tissues of endometriosis.^[87]^ In contrast, TC damage in the fallopian tube may lead to impaired immunomodulation/immunosurveillance, diminished intercellular signaling and tubal constriction.

Inflammation and ischemia can cause extensive ultrastructural damage to TC cell bodies and TPs, with significant TC loss and interstitial fibrotic remodeling. Such pathological changes may lead to structural and functional abnormalities of the tubal tissue and even to infertility. In normal and diseased tissues, TCs are linked to various activated immune cells that may be involved in local immune regulation (suppression or activation) and may be a cause of immune-mediated pregnancy failure. An in vitro TC study has shown that TCs can activate peritoneal macrophages in mice and subsequently trigger the secretion of inflammatory cytokines, among other factors, suggesting that TCs are not merely innocent bystanders, but potential participants in local immune regulation and immune surveillance.^[86]^

## 5. Discussion

Telocytes, novel mesenchymal cells, are expressed in almost all tissues and organs. TCs have important functions such as mechanical support, signaling, immune regulation, and tissue repair. They not only participate in the balance of tissue homeostasis, but also participate in the pathophysiological process and tissue repair and regeneration of various diseases. In particular, inflammatory diseases and malignant tumor-related diseases have been widely studied in recent years. TCs play a vital role in some inflammatory diseases and may become a new choice for the treatment of inflammatory diseases. The experimental study of TCs is not yet deep enough and has been almost at a relative standstill in recent years, and many of the functions assigned to TCs do not originate from direct functional experimental validation. It is hoped that future studies will continue to deepen and find specific biological molecular markers of TCs, in order to better investigate its key role in physiopathological processes. In the future, we will explore the role of TCs in the development of ulcerative colitis and its mechanism of action, which may provide new ideas for the treatment of ulcerative colitis.

## Author contributions

**Resources:** Hu Tian.

**Writing – review & editing:** Yuhua Zhang.
